# Pax6 is required intrinsically by thalamic progenitors for the normal molecular patterning of thalamic neurons but not the growth and guidance of their axons

**DOI:** 10.1186/s13064-015-0053-7

**Published:** 2015-10-31

**Authors:** James M. Clegg, Ziwen Li, Michael Molinek, Isabel Martín Caballero, Martine N. Manuel, David J. Price

**Affiliations:** Centre for Integrative Physiology, University of Edinburgh, Hugh Robson Building, George Square, Edinburgh, EH8 9XD UK; Current address: Laboratory of Molecular Neurobiology, Karolinska Institute, 17177 Scheeles Väg 1, Sweden

**Keywords:** Nkx2.2, Pax6, Robo2, Thalamic patterning, Thalamocortical axons, Transcription factors

## Abstract

**Background:**

In mouse embryos, the Pax6 transcription factor is expressed in the progenitors of thalamic neurons but not in thalamic neurons themselves. Its null-mutation causes early mis-patterning of thalamic progenitors. It is known that thalamic neurons generated by *Pax6*^*−/−*^ progenitors do not develop their normal connections with the cortex, but it is not clear why. We investigated the extent to which defects intrinsic to the thalamus are responsible.

**Results:**

We first confirmed that, in constitutive *Pax6*^*−/−*^ mutants, the axons of thalamic neurons fail to enter the telencephalon and, instead, many of them take an abnormal path to the hypothalamus, whose expression of Slits would normally repel them. We found that thalamic neurons show reduced expression of the Slit receptor *Robo2* in *Pax6*^*−/−*^ mutants, which might enhance the ability of their axons to enter the hypothalamus. Remarkably, however, in chimeras comprising a mixture of *Pax6*^*−/−*^ and *Pax6*^*+/+*^ cells, *Pax6*^*−/−*^ thalamic neurons are able to generate axons that exit the diencephalon, take normal trajectories through the telencephalon and avoid the hypothalamus. This occurs despite abnormalities in their molecular patterning (they express Nkx2.2, unlike normal thalamic neurons) and their reduced expression of *Robo2*. In conditional mutants, acute deletion of Pax6 from the forebrain at the time when thalamic axons are starting to grow does not prevent the development of the thalamocortical tract, suggesting that earlier extra-thalamic patterning and /or morphological defects are the main cause of thalamocortical tract failure in *Pax6*^*−/−*^ constitutive mutants.

**Conclusions:**

Our results indicate that Pax6 is required by thalamic progenitors for the normal molecular patterning of the thalamic neurons that they generate but thalamic neurons do not need normal Pax6-dependent patterning to become competent to grow axons that can be guided appropriately.

## Background

The highly conserved transcription factor Pax6 is required for normal thalamic development [1–10]. It is expressed in thalamic progenitor cells at the earliest stages of diencephalic development, is later progressively downregulated in these progenitors and is not expressed in postmitotic thalamic neurons [3, 11–14]. One of its early functions is to ensure the normal molecular patterning of thalamic progenitors by repressing the expression of *Shh* [10]. In its absence, thalamic neurons are produced, albeit in reduced numbers, and they retain an expression profile similar in many respects to that of normal thalamic neurons [2–7]. Pax6 is, however, required for the development of thalamocortical axons (TCAs), which connect the thalamus to the cortex [6, 7, 15–17]. The reasons for this are poorly understood and might lie inside or outside the thalamus. Pax6 is expressed not only in the thalamus but also by cells in extra-thalamic diencephalic and ventral telencephalic regions through which TCAs normally grow and in the cerebral cortex itself; this extra-thalamic expression is contemporaneous with TCA formation [6, 13, 18]. Pinon et al. [19] used conditional mutants to generate a cortex-specific deletion of Pax6 and found that Pax6 is not required by cortical cells for TCA development. Previous studies have not tested whether Pax6 is required cell autonomously by thalamic neurons for the development of their axons. We set out to compare axonal development in *Pax6*^*−/−*^ thalamic neurons in constitutive *Pax6*^*−/−*^ mutants and in *Pax6*^*−/−*^*↔Pax6*^*+/+*^ chimeras to discover whether the axons of mutant thalamic neurons have the competence to grow appropriately.

In mouse embryos, TCAs begin to grow at about embryonic day 12.5 (E12.5). They extend rostro-ventrally through the adjacent prethalamus before turning laterally, away from the hypothalamus, to enter the ventral telencephalon by E13.5. After crossing the ventral telencephalon, they turn dorsally into the developing cortex. They reach the correct regions of the cortex by about E18.5 [16, 20–22]. The mechanisms that guide these axons are likely to include both positive and negative cues, guiding the axons towards correct targets or away from incorrect targets respectively. There is evidence that the positive cues include early pioneer axons originating in the ventral telencephalon that grow to the thalamus and provide guidance for thalamic axons on the first part of their journey towards the cortex [23–26]. Data on the timing of the development of these ventral telencephalic projections combined with evidence that mutant mice showing absence, shrinkage or displacement of this population also show defective TCA extension into the ventral telencephalon are consistent with the idea that ventral telencephalic projections might act as a scaffold [27]. Previous studies have shown defects of these projections in *Pax6*^*−/−*^ embryos that might go at least some way towards explaining the TCA defects in these mutants [21, 28, 29].

Regarding the molecular cues that guide TCA development, the guidance receptors Robo 1 and 2 and their ligands Slit 1 and 2 [30] have been shown to play important roles [31–36]. There is evidence that negative cues operate as thalamic axons exit the prethalamus, at which point they turn sharply laterally away from the hypothalamus in the direction of the diencephalic-telencephalic border. Several studies have shown that: (i) hypothalamic explants repel thalamic axons in explant cultures [7, 35]; (ii) the hypothalamus expresses high levels of Slits, which are generally chemorepellent for growing axons, and thalamic axons express the Robo receptors through which they signal; (iii) in both *Slit2*^*−/−*^ and *Slit1*^*−/−*^;*Slit2*^*−/−*^ mutants, a large number of thalamic projections fail to enter the telencephalon and instead descend into the hypothalamus [26, 31, 34, 35]. These findings provide compelling evidence that Slit-mediated repulsion contributes to the deflection of thalamic axons away from the hypothalamus and across the diencephalic-telencephalic boundary.

Here, we found that thalamic neurons show reduced expression of the Slit receptor *Robo2* in *Pax6*^*−/−*^ mutants, which might enhance the ability of their axons to enter the hypothalamus. In chimeras comprising a mixture of *Pax6*^*−/−*^ and *Pax6*^*+/+*^ cells, *Pax6*^*−/−*^ thalamic neurons were able to generate axons that exit the diencephalon, take normal trajectories through the telencephalon and avoid the hypothalamus, despite abnormalities in their molecular patterning. Our findings indicate that Pax6 is required by thalamic progenitors for the normal molecular patterning of the thalamic neurons that they generate but thalamic neurons do not need normal Pax6-dependent patterning to become competent to grow axons that can be guided appropriately.

## Results

### Severe thalamic axonal pathfinding defects in *Pax6*^*−/−*^ mice

**Fig. 1:**
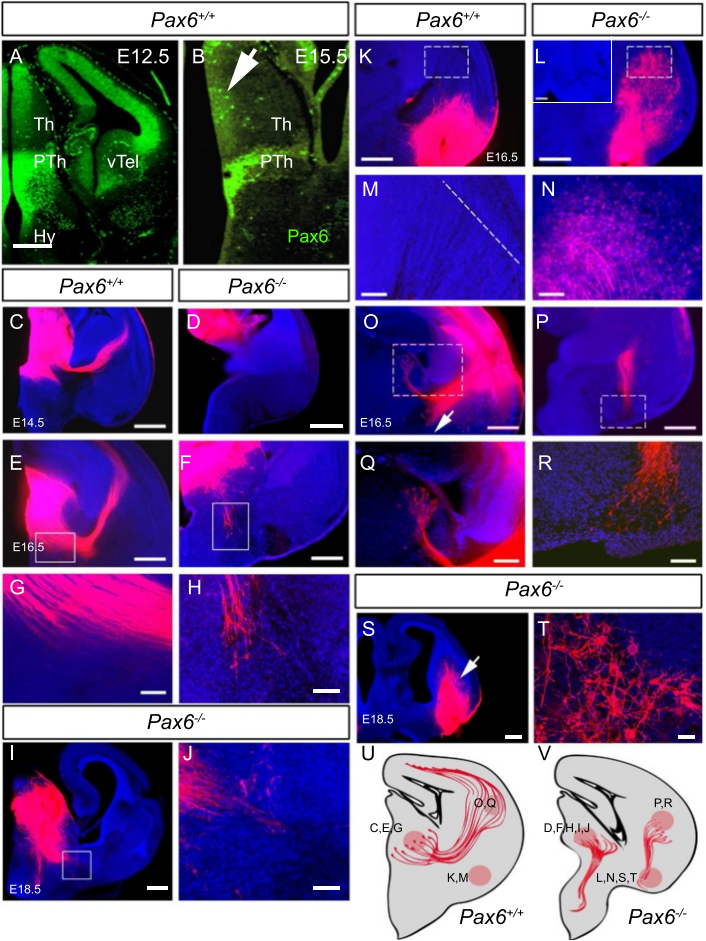
TCA pathfinding defects in *Pax6* ^*−/−*^ mice. **a**, **b** Normal expression of Pax6 shown with immunohistochemistry at E12.5 and E15.5; Th, thalamus; PTh, prethalamus; Hy, hypothalamus; vTel, ventral telencephalon. Arrow indicates residual Pax6 staining in the ventricular zone at E15.5. **c**, **e** DiI placement in the thalamus of WT mice reveals TCAs extending through the ventral telencephalon at E14.5 and entering the cortex in greater numbers by E16.5. **d**, **f** DiI placement in the thalamus of *Pax6* ^*−/−*^ mice shows no axons leaving the thalamus at E14.5; at E16.5 a small number of thalamic axons can be seen heading towards the hypothalamus, while no axons are observed in the ventral telencephalon (**f**). **g**, **h** Boxed areas in e,f at higher magnification. **i**, **j** DiI placement in the thalamus of *Pax6* ^*−/−*^ mice shows the failure of axons to leave the diencephalon at E18.5; boxed area in I is shown in j. **k**, **m** DiI placement close to the amygdaloid region in *Pax6* ^*+/+*^ mice does not label any major axon tract whereas (**l**, **n**) in *Pax6* ^*−/−*^ mice it labels a large axon tract within the ventral telencephalon and a large number of cell bodies close to the PSPB (n). Inset in l shows the lack of DiI labelling within the thalamus, demonstrating that the labelled axons are not of thalamic origin. Boxed areas in k,l are shown at higher magnification in m,n; broken line in M = PSPB. **o**, **q** DiI placement close to the PSPB in *Pax6* ^*+/+*^ mice labels the thalamocortical tract (**o**), cell bodies within the thalamus (q) and axons descending to sub-striatal targets (arrow in o). **p**, **r** In *Pax6* ^*−/−*^ mice it reveals a large bundle of axons that extend ventrally (p) and are tipped with growth cones (**r**). Boxed areas in (**o**, **p**) are shown at higher magnification in (**q**, **r**). **s**, **t** DiI placement close to the amygdaloid region in *Pax6* ^*+/+*^ mice gave similar results at E18.5 to those at E16.5. **u**, **v** Schematic diagrams summarising the axon pathfinding defects observed in *Pax6* ^*−/−*^ mice (**v**) compared to *Pax6* ^*+/+*^ mice (**u**). DiI injections sites with relevant panels are marked. Scale bars: **a**–**f**, **i**, **k**, **l**, **o**, **p**, **s** 500 μm, **g**, **h**, **j**, **m**, **n**, **q**, **r** 100 μm, **t** 10 μm

In the normal mouse diencephalon at E12.5, when TCAs are starting to grow, Pax6 is expressed by progenitor cells in the thalamus and by both progenitors and postmitotic neurons in the prethalamus (Fig. 1a). Pax6 is not expressed by postmitotic neurons in the thalamus. The thalamic postmitotic layer expands over the following days, leaving only a few Pax6+ cells at the ventricular edge (arrow in Fig. 1b). Postmitotic Pax6+ cells persist in the prethalamus (Fig. 1b).

We used the carbocyanine dye, DiI, to label axons exiting the thalamus in wild-type (WT) and *Pax6*^*−/−*^ brains from E14.5-18.5. Whereas axons extended from the thalamus through the ventral telencephalon and were crossing the pallial-subpallial boundary (PSPB) in E14.5 WT brains (Fig. 1c), no such axons were labelled in *Pax6*^*−/−*^ brains (Fig. 1d). In E16.5 WT brains, many more TCAs extended across the PSPB to reach the cortex (Fig. 1e), but in *Pax6*^*−/−*^ brains only small numbers of axons extended from the thalamus and, rather than crossing into the ventral telencephalon, they invaded the hypothalamus (Fig. 1f–h), a region normally repulsive to thalamic axons [7, 35]. Thalamic axons had made no further progress into the ventral telencephalon at E18.5 in *Pax6*^*−/−*^ embryos (Fig. 1i, j) (note that *Pax6*^*−/−*^ embryos do not survive for a significant time after birth).

**Fig. 2:**
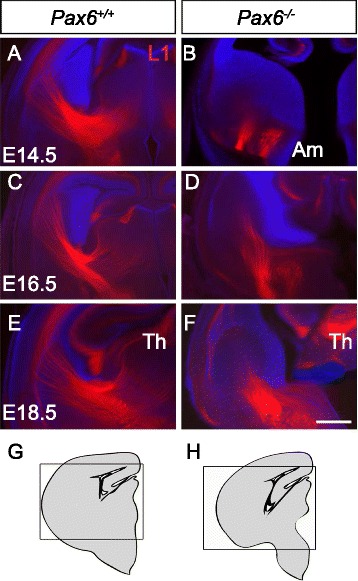
L1 labelled forebrain axonal tracts are abnormal in *Pax6* ^*−/−*^ embryos. Immunohistochemistry for axonal marker L1 labels axonal tracts at E14.5 (**a**, **b**), E16.5 (**c**, **d**) and E18.5 (**e**, **f**). In *Pax6* ^*+/+*^ embryos TCAs and corticofugal axons can be observed running between the thalamus and cortex (**a**, **c**, **e**). In *Pax6* ^*−/−*^ embryos L1 labels an axon tract within the ventral telencephalon which increases in size between E14.5 and E18.5 (**b**, **d**, **f**). **g**, **h** Boxes mark the approximate areas shown in (**a**–**f**). Scale bar: 500 μm

L1 immunohistochemistry was used to label ascending axons from the thalamus and descending axons from the cortex between E14.5 and E18.5 since both TCAs and corticofugal axons express L1 throughout this period [15]. Fig. 2a, c, e shows L1+ axons of these tracts in *Pax6*^*+/+*^ brains between E14.5 and E18.5. In *Pax6*^*−/−*^ brains, we observed large bundles of L1-labelled axons within the ventral telencephalon close to the amygdaloid region at E14.5 and extending between this region and the PSPB by E16.5 and E18.5 (Fig. 2b, d, f). We investigated these axons further with DiI labelling to discover their origin.

DiI was placed at each end of the *Pax6*^*−/−*^ ventral telencephalic L1+ tract, either (i) in the amygdaloid region in the ventral aspect of the ventral telencephalon, or (ii) close to the PSPB. DiI in the amygdaloid region (ventral to the internal capsule) did not label any long-range axonal tract to or from this area in WT brains (Fig. 1k, m), but in *Pax6*^*−/−*^ brains it labelled an axon tract running between it and the PSPB (Fig. 1l, n, s, t). Numerous cell bodies were retrogradely labelled around the PSPB (mainly ventral to it) (Fig. 1l, n, t) but none were labelled in the thalamus (Fig. 1l). In WT brains, DiI placement at the PSPB labelled axons passing through or originating in this region, including (i) thalamocortical axons and their cell bodies in the thalamus, (ii) corticofugal axons with their cell bodies in the cortex and (iii) striatal neurons with their cell bodies at the injection site and axons projecting to sub-striatal targets in the substantia nigra (Fig. 1o, q). In *Pax6*^*−/−*^ brains, DiI at the PSPB only labelled axons tipped with growth cones extending towards the amygdaloid region (Fig. 1p, r).

These experiments reveal an aberrant axon tract in the *Pax6*^*−/−*^ ventral telencephalon. It originates from cells close to the PSPB that project axons in a ventral direction towards the amygdaloid region and is not connected to the thalamus. It is possible that this is a misrouted striatonigral pathway [37]. Our results indicate that many axons leaving the thalamus grow to the hypothalamus but not the ventral telencephalon, as summarized in Fig. 1(u, v).

### *Robo2* expression is reduced in the *Pax6*^*−/−*^ thalamus

We considered the question of why *Pax6*^*−/−*^ thalamic axons failed to avoid the hypothalamus in *Pax6*^*−/−*^ mutants. Since *Slit1* and *Slit2* and *Robo1* and *Robo2* normally provide cues that steer TCAs away from the hypothalamus [31, 33, 35], we examined their expression by in situ hybridisation and quantitative reverse-transcription PCR (qRT-PCR) in WT and *Pax6*^*−/−*^ mice.

**Fig. 3:**
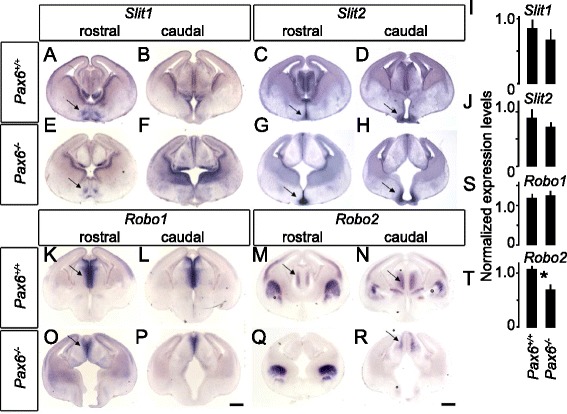
Hypothalamic *Slit* expression is maintained while thalamic *Robo2* expression is reduced in *Pax6* ^*−/−*^ embryos at E13.5. **a**–**d** In situ hybridisation at E14.5 shows *Slit1* and *Slit2* mRNA expression at the hypothalamus in *Pax6* ^*+/+*^ embryos (arrows, a,c,d). **e**–**h** In situ hybridisation shows that *Slit1* and *Slit2* mRNA expression is maintained in the hypothalamus in *Pax6* ^*−/−*^ embryos (arrows **e**, **g**, **h**). **i**, **j** Quantitative RT-PCR on tissue from the hypothalamus shows no significant difference in *Slit1* (**i**) and *Slit2* (**j**) mRNA expression between *Pax6* ^*+/+*^ and *Pax6* ^*−/−*^ embryos (Student’s t-tests, *n* = 5 WT and 5 mutants). **k**–**n** In situ hybridisation for *Robo1* and *Robo2* shows mRNA expression of both genes within the thalamus of *Pax6* ^*+/+*^ embryos (arrows k,m,n). **o**–**r** In situ hybridisation in *Pax6* ^*−/−*^ embryos shows that *Robo1* expression is still present within the thalamus although the expression domain appears reduced in size (arrow **o**). In situ hybridisation for *Robo2* shows that the expression is reduced within the thalamus of *Pax6* ^*−/−*^ embryos, particularly at rostral levels (**q**). **s**, **t** Quantitative RT-PCR performed on the whole thalamus shows that while there is no difference in *Robo1* expression between *Pax6* ^*+/+*^ and *Pax6* ^*−/−*^ embryos (**s**; Student’s *t*-test, *n* = 5 WT and 5 mutants), *Robo2* mRNA expression is significantly reduced in *Pax6* ^*−/−*^ embryos compared to *Pax6* ^*+/+*^ embryos (**t**; Student’s *t*-test, *n* = 5 WT and 5 mutants, *p* = 0.03). Scale bars: 500 μm

*Slit1* and *Slit2* are expressed in the hypothalamus of WT and *Pax6*^*−/−*^ embryos at E13.5 (Fig. 3a–h). To quantify the level of *Slit* mRNA expression we performed quantitative RT-PCR using tissue from the hypothalamus: we found no significant differences in the levels of *Slit1* or *Slit2* expression between *Pax6*^*−/−*^ and WT mice (Fig. 3i, j). In the E13.5 WT, *Robo1* is expressed in the thalamus, close to the ventricular zone at the midline (Fig. 3k, l). *Robo2* is expressed in the body of the thalamus, where the differentiating neurons are located (Fig. 3m, n). In the E13.5 *Pax6*^*−/−*^ mouse, *Robo1* is expressed in a similar location to that in WTs (Fig. 3o,p). Although the expression domain of *Robo1* is reduced in size, qRT-PCR using tissue from the thalamus showed no significant difference in the levels of *Robo1* expression between *Pax6*^*−/−*^ and WT mice (Fig. 3s), indicating that the reduction in expression domain is in proportion to the reduction in the size of the thalamus that occurs in these mutants. The level of *Robo2* expression within the E13.5 *Pax6*^*+/+*^ thalamus, however, showed a significant overall reduction (Student’s *t*-test, *n* = 5 WT and 5 mutants, *p* = 0.03; Fig. 3t). The reduction appeared greatest in rostral thalamus (Fig. 3q,r). A reduction in *Robo2* expression by *Pax6*^*−/−*^ thalamic neurons might contribute to their axons’ abnormal invasion of the hypothalamus.

### Cell autonomous defects of gene expression in *Pax6*^*−/−*^ thalamic neurons

**Fig. 4:**
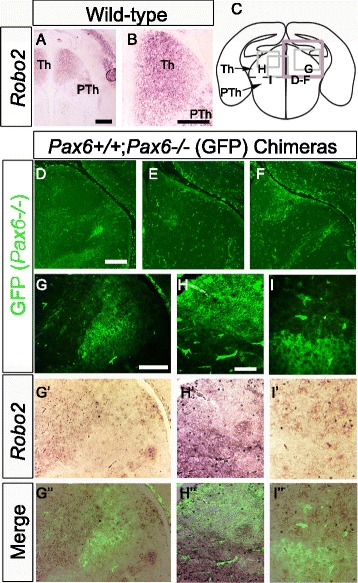
Reduced *Robo2* expression by *Pax6* ^*−/−*^ thalamic neurons in chimeras. **a,b** *Robo2* expression in wild-type thalamus at E16.5. **c** Diagram shows the positions of the thalamic areas photographed in **d**–**i**”; Th, thalamus; PTh, prethalamus. **d**–**f** Examples of the contributions of *Pax6* ^*−/−*^ (GFP+) cells to the thalamus of three chimeras. **g**–**i**” Examples showing more intense staining for *Robo2* mRNA (brown/purple) in WT cells than in clumps of GFP+ *Pax6* ^*−/−*^ cells in the thalamus of a chimera aged E16.5. Scale bars: A,B, 100 μm; **d**–**f**, 75 μm; **g**–**i**”, 50 μm

The findings described above indicate major defects of thalamic neurons in *Pax6*^*−/−*^ embryos. We tested whether *Pax6*^*−/−*^ thalamic neurons show defects, first of gene expression and later of axonal growth, in *Pax6*^*−/−*^↔*Pax6*^*+/+*^ chimeras comprising a majority of *Pax6*^*+/+*^ cells. *Pax6*^*−/−*^ cells in chimeras were marked by a GFP transgene (Fig. 4). Defects detected in mutant neurons surrounded by a majority of WT cells are more likely to be caused by mechanisms intrinsic to the *Pax6*^*−/−*^ lineage. Figure 4d–f shows images from three different chimeras taken from the region outlined in purple in Fig. 4c: consistently, most neurons in the thalamus of our chimeras were WT.

Figure 4a, b shows that *Robo2* is normally expressed throughout the WT embryonic thalamus. In chimeras, *Pax6*^*−/−*^ thalamic neurons showed reduced *Robo2* expression compared to their WT neighbours (Fig. 4g–i”; data are from three different regions of the thalamus of the same chimera, outlined in grey in Fig. 4c). This suggested that their reduced expression of *Robo2* is a direct consequence of the absence of Pax6 from their progenitors.

**Fig. 5:**
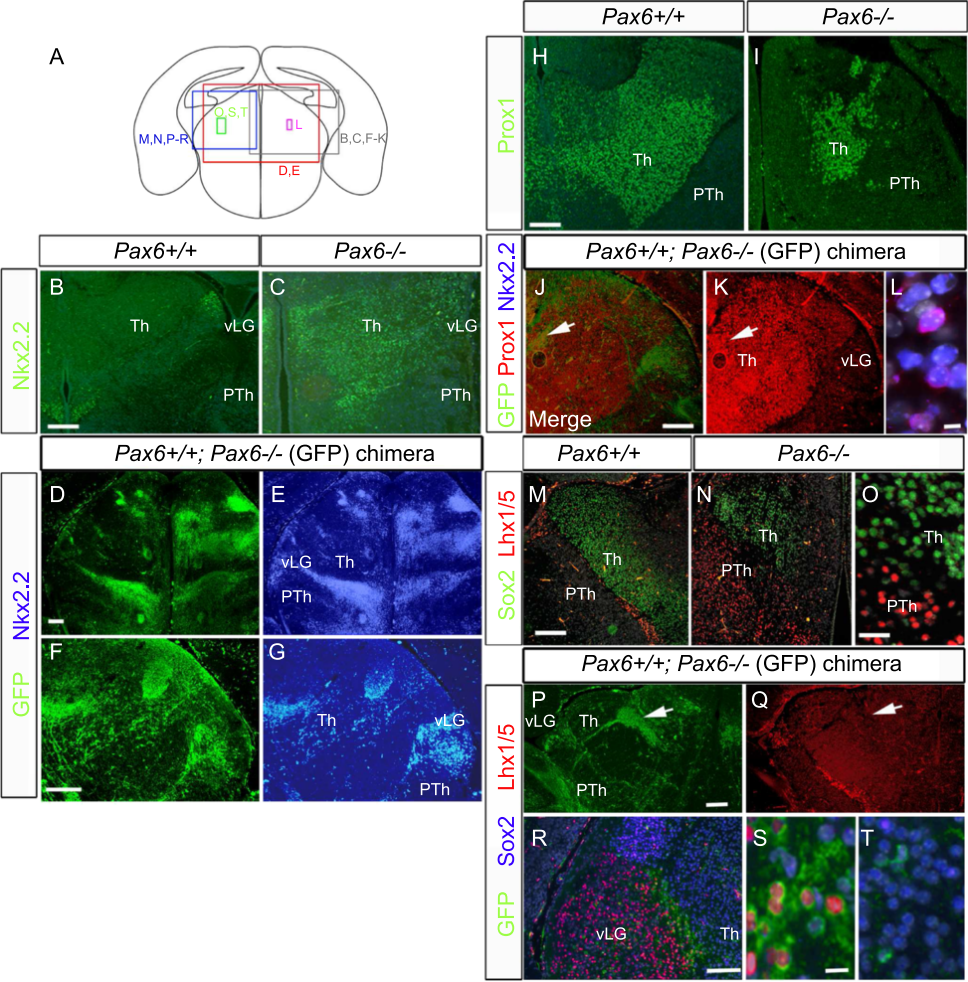
Abnormal upregulation of Nkx2.2 by *Pax6* ^*−/−*^ neurons in chimeras. **a** Diagram showing the locations of the panels in the rest of the Figure. **b,c** Nkx2.2 protein expression in (**b**) WT and (**c**) *Pax6* ^*−/−*^ embryos at E16.5. Expression is normally confined to a few cells along the border of thalamus (Th) and prethalamus (PTh) and the vLG (**b**) but is expressed widely throughout the *Pax6* ^*−/−*^ thalamus (c). **d**–**g** Nkx2.2 is expressed by *Pax6* ^*−/−*^ GFP+ cells, but not by WT cells, throughout the thalamus in E16.5 chimeras. **h**, **i** Prox1 protein expression in (**h**) *Pax6* ^*+/+*^ and (**i**) *Pax6* ^*−/−*^ embryos at E16.5. Prox1 is expressed in the WT thalamus and in the mutant thalamus, which is reduced in size. **j**, **k** *Pax6* ^*−/−*^ GFP+ cells in the thalamus (e.g. arrow in **j**) do not show abnormal expression of Prox1. **l** Many of the *Pax6* ^*−/−*^ neurons that express (abnormally) Nkx2.2 in the thalamus of chimeras co-express Prox1. **m**, **n**, **o** Sox2 and Lhx1/5 protein expression in (**m**) WT and (n) *Pax6* ^*−/−*^ embryos at E16.5. In both genotypes, Sox2 is confined to the thalamus, which does not co-express Lhx1/5. **p**, **q** *Pax6* ^*−/−*^ GFP+ cells in the thalamus (e.g. arrow) do not show abnormal expression of Lhx1/5, which is expressed as normal along the thalamic-prethalamic border and in the vLG. **r**–**t** *Pax6* ^*−/−*^ GFP+ cells show normal expression of Lhx1/5 in the vLG and of Sox2 in the thalamus (**r**); *Pax6* ^*−/−*^ GFP+ cells close to the border express either one or other of these markers but not both (**s**); *Pax6* ^*−/−*^ GFP+ thalamic cells express Sox2, as do their WT neighbours (**t**). Scale bars: **b**-**k**, **m**, **n**, **p**, **q**, 50 μm; L, 5 μm; O, 15 μm; R, 25 μm; S,T, 10 μm

We also examined the expression of markers of diencephalic patterning in chimeras (Fig. 5). These markers were: (i) Nkx2.2, whose expression is normally restricted to domains around the border between thalamus and prethalamus including the ventral lateral geniculate nucleus (vLG) but expands throughout the thalamus in *Pax6*^*−/−*^ embryos (Fig. 5b, c); (ii) Prox1 and Sox2, which retain their expression in the thalamus of *Pax6*^*−/−*^ embryos (Fig. 5h, i, m–o) [2, 10]; (iii) Lhx1/5, whose expression is normally restricted to the boundary of thalamus and prethalamus and the vLG (Fig. 5m) and expands through the prethalamus but not the thalamus of *Pax6*^*−/−*^ mutants at E16.5 (Fig. 5n, o). The most striking abnormality in chimeras was expression of Nkx2.2 throughout the thalamus by *Pax6*^*−/−*^ neurons (Fig. 5d–g). Clusters of mutant cells in chimeras did not show any obvious signs of losing Prox1 expression (Fig. 5j, k). As a consequence, many Nkx2.2, Prox1 double-labelled mutant cells were identified throughout the thalamus (Fig. 5l). Such cells would never normally be present since the domains of Prox1 and Nkx2.2 do not normally overlap. *Pax6*^*−/−*^ neurons did not upregulate Lhx1/5 in the thalamus of chimeras (Fig. 5p, q). Where mutant cells were located close to the border of thalamus and prethalamus, they expressed either Lhx1/5 or Sox2 but not both (Fig. 5r, s). *Pax6*^*−/−*^ thalamic cells expressed Sox2, as did their WT neighbours (Fig. 5t). These findings suggest that *Pax6*^*−/−*^ thalamic neurons in chimeras are partially mis-patterned to express an abnormal combination of transcription factors, some of which they would normally express and at least one of which they would not.

### Axons of *Pax6*^*−/−*^ thalamic neurons follow a normal trajectory in chimeras

**Fig. 6:**
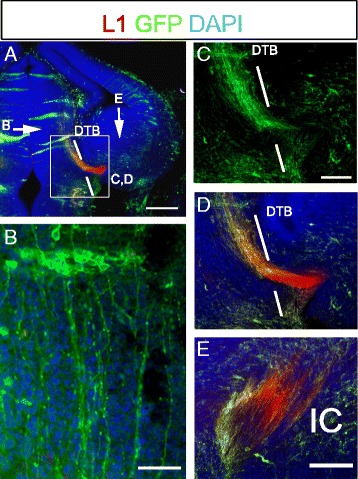
*Pax6* ^*−/−*^ thalamic axons can exit the diencephalon in *Pax6* ^*−/−*^ ↔Pax6^+/+^ chimeras. GFP staining shows tauGFP *Pax6* ^*−/−*^ axons arising from small clusters of mutant cells in the thalamus: in (**a**), arrows mark the positions of regions imaged in slightly different planes of section in panels (**b**) and (**e**) and the boxed area is shown in panels (**c**) and (**d**). GFP+ *Pax6* ^*−/−*^ axons were able to exit the thalamus and enter the internal capsule (IC) with a trajectory that overlapped the normal L1+ thalamocortical tract. DTB: diencephalic-telencephalic border. Scale bars: A, 500 μm; B, 50 μm; C,D, 100 μm; E, 75 μm

In *Pax6*^*−/−*^↔*Pax6*^*+/+*^ chimeras, *Pax6*^*−/−*^ cells were labelled with tauGFP, allowing their axons to be visualized. Chimeras were studied at E13.5, which is before corticofugal axons have extended across the ventral telencephalon to the thalamus, so avoiding potential confusion between GFP+ thalamic axons and GFP+ corticofugal axons. By E13.5, WT TCAs have turned into the ventral telencephalon and generated the internal capsule but have not yet reached the PSPB [21]. As for the gene expression analysis, we examined chimeric embryos in which the majority of cells were *Pax6*^*+/+*^ (Fig. 6a).

We observed that tauGFP *Pax6*^*−/−*^ axons arising from small clusters of mutant cells in the thalamus (Fig. 6b) were able to exit the thalamus (Fig. 6c, d) and traverse the internal capsule with a trajectory that overlapped the normal L1+ thalamocortical tract in chimeras (Fig. 6e). There was no evidence of an abnormal projection of mutant cells to the hypothalamus. This remarkable rescue indicates that thalamic cells do not have a cell autonomous inability to generate axons that can follow a normal route out of the diencephalon. Their intrinsic molecular defects described above are insufficient to prevent their axons from being guided into the telencephalon by surrounding WT cells and their axons.

### Pax6 loss at the time of thalamic axonal growth does not prevent TCA formation

**Fig. 7:**
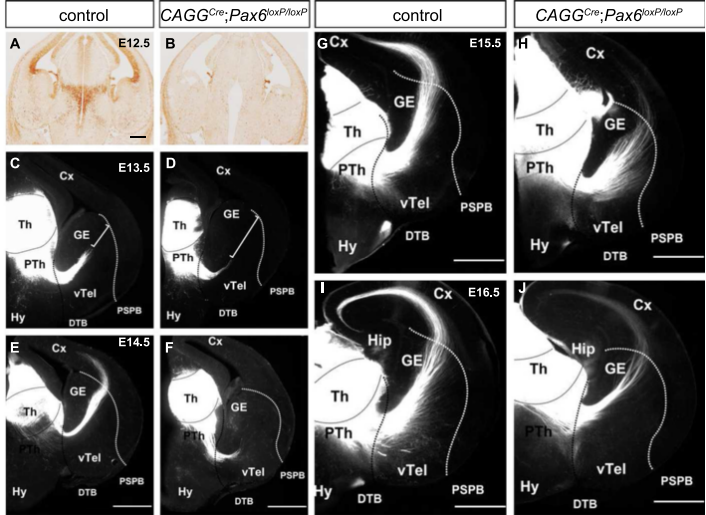
Loss of Pax6 expression at E12.5 in *CAGG* ^*Cre*^; *Pax6* ^*loxP/loxP*^ embryos delays thalamic axonal growth. **a**, **b** Pax6 immunohistochemistry shows a loss of Pax6 expression at E12.5 throughout the brain in *CAGG* ^*Cre*^; *Pax6* ^*loxP/loxP*^ embryos compared to controls. **c**–**j** DiI placement in the thalamus (Th) labels TCAs extending through the prethalamus (PTh), avoiding the hypothalamus (Hy) and crossing the ventral telencephalon (vTel) to the cortex (Cx) in both control and *CAGG* ^*Cre*^; *Pax6* ^*loxP/loxP*^ embryos between E13.5 and E16.5. **c**, **d** At E13.5, most axons in the control cross the diencephalic-telencephalic boundary (DTB) and are half way through the ventral telencephalon, whereas in *CAGG* ^*Cre*^; *Pax6* ^*loxP/loxP*^ embryos, the advancement of axons is delayed (white bar shows the distance between the tips of the TCAs and the pallial-subpallial boundary [PSPB]). GE = ganglionic eminence. **e**, **f** A large number of axons in the control have crossed the PSPB at E14.5 but the axons in *CAGG* ^*Cre*^; *Pax6* ^*loxP/loxP*^ embryos remain in the ventral telencephalon. **g**, **h** In control embryos at E15.5 TCAs navigate further into the cortex whereas in *CAGG* ^*Cre*^; *Pax6* ^*loxP/loxP*^ embryos some axons just cross the PSPB. **i**, **j** By E16.5 TCAs have reached the cortex in both *CAGG* ^*Cre*^; *Pax6* ^*loxP/loxP*^ and control embryos. Hip = hippocampus. Scale bars, 500 μm

Since Pax6 is not required to generate thalamic neurons with the competence to grow axons that can be guided appropriately, the failure of TCA development in constitutive *Pax6*^*−/−*^ mutants is presumably caused by defects extrinsic to thalamic neurons and/ or their axons. Cells in the environment of thalamic neurons and/ or their axons might need Pax6 at the time when TCAs are forming to provide adequate molecular/ morphological support. Alternatively, they might have needed Pax6 before TCAs start forming to establish the correct conditions. To distinguish between these two possibilities, we conditionally inactivated *Pax6* using tamoxifen administered at E9.5 to *Pax6*^*loxP*^ embryos ubiquitously expressing Cre^ER^ from the *CAGG-Cre*^*ER*^ allele [18, 38]. Tamoxifen-treated experimental embryos carried two copies of the floxed allele (*CAGG*^*Cre*^; *Pax6*^*loxP/loxP*^), those carrying only one copy were controls (TCAs are unaffected in *Pax6*^*+/−*^ embryos). Loss of Pax6 protein, which was complete before E12.5 (Fig. 7a, b), coincided with the onset of TCA growth [16, 20–22]. By E12.5, the loss of Pax6 had already caused some morphological changes characteristic of constitutive *Pax6*^*−/−*^ mutants, including expansion of the 3^rd^ ventricle, that were less severe than those in constitutive *Pax6*^*−/−*^ mutants of comparable age (Fig. 7a, b).

Deleted embryos were examined at E13.5-E16.5 (Fig. 7c–j). They did not show the severe TCA defects found in *Pax6*^*−/−*^ constitutive mutants. The thalamocortical tract formed, although its growth was ~1–2 days behind that in controls. These results strengthen greatly the probability that the devastating effect of constitutive Pax6 loss on TCA development is a relatively indirect consequence of patterning and/or morphological defects that occur before TCAs start to form.

## Discussion

Our key finding is that, while Pax6 is required cell autonomously by thalamic progenitors for their neuronal progeny to develop a correct molecular profile, it is not required for thalamic neurons to develop the competence to grow axons that can be guided correctly out of the thalamus and towards the cortex. This is in striking contrast to the inability of thalamic axons to exit the diencephalon in constitutive *Pax6*^*−/−*^ mutants, a finding in line with several previous reports [6, 15, 17]. This result is important because it rules out the possibility that *Pax6*^*−/−*^ thalamic neurons are intrinsically unable to grow axons of sufficient length to exit the diencephalon.

This major rescue of the *Pax6*^*−/−*^ mutant phenotype presumably results from the restoration of elements critical for normal TCA development by the *Pax6*^*+/+*^ cells in the chimeras. By allowing Pax6 expression during early forebrain development and then removing it ubiquitously as TCAs start to form we showed that the Pax6-dependent elements required for TCA navigation are in place before the tract forms. There are several strong non-exclusive possibilities as to what the Pax6-dependent elements might be. First, developing thalamic axons might require Pax6-dependent signals from other thalamic axons, for example those they fasciculate with, to exit the diencephalon; these signals would be restored in chimeras but would be absent in constitutive mutants. Second, Pax6 is required for early patterning and morphological development of the extra-thalamic diencephalon and ventral telencephalon, regions through which TCAs must navigate. Defects of these regions, which are severe in *Pax6*^*−/−*^ constitutive mutants [2–7, 10] but are ameliorated in chimeras, might prevent TCA development in constitutive mutants.

The first tissue that thalamic axons encounter as they exit the thalamus is the prethalamus. Previous studies have shown the importance of the prethalamus for TCA development [16, 39, 40] and Pax6 is critical for its normal patterning [2, 5, 10]. Although thalamic axons are able to cross the prethalamus in constitutive *Pax6*^*−/−*^ mutants, it is possible that a Pax6-dependent interaction of prethalamic cells with thalamic axons might confer on thalamic axons the ability to navigate into and through the telencephalon. Constitutive *Pax6*^*−/−*^ embryos always show substantial narrowing and anatomical distortion of the diencephalic-telencephalic junction through which thalamic axons would normally navigate [4, 5, 7]. These defects, which were not present in our chimeras, provide an obvious mechanical explanation for the extremely severe thalamic axonal defects in constitutive *Pax6*^*−/−*^ embryos. A loss of the ventral telencephalic pioneer axons that are hypothesized to guide developing TCAs into the ventral telencephalon [23–26] might also precipitate a failure of the thalamocortical tract in *Pax6*^*−/−*^ mutants. The effects of a transcription factor such as Pax6 on a complex process such as the development of TCAs, which involves multiple tissues that express the transcription factor for many days before the axons grow, are likely to be numerous. It seems probable, therefore, that multiple Pax6-loss-induced defects conspire to prevent thalamic axons from expressing their competence to grow correctly.

Interestingly, we found that the cell autonomous molecular defects of *Pax6*^*−/−*^ thalamic do not prevent them developing the competence to grow axons correctly. Their reduced expression of *Robo2* in chimeras was insufficient to cause them to navigate incorrectly to the hypothalamus. This might be because the magnitude of the reduction was not large enough to tip the balance of competing influences in favour of this outcome in chimeras. The fact that *Pax6*^*−/−*^ thalamic neurons show strong activation of *Nkx2.2*, in constitutive mutants and chimeras, suggests that this particular transcription factor does not interfere with these neurons’ competence to develop axons that grow correctly. In the spinal cord, where Pax6 deletion also causes an expansion in the domain of Nkx2.2 expression, Nkx2.2 plays an important role in determining whether neurons develop as projection neurons or interneurons and to the regulation of programs generating specific types of motor neurons [41, 42]. It is possible that the effects of its ectopic Pax6-loss-induced expression in the thalamus are curtailed by the maintenance of relative normality in the expression of other patterning transcription factors.

Like *Nkx2.2*, regulation of *Robo2* by Pax6 is not confined to the thalamus. Previous work has indicted that it also occurs in the cerebral cortex [43]. Indeed, this can be observed in *Pax6*^*−/−*^ mutants in Fig. 3(m, n, q, r): Robo2 expression is reduced in the cerebral cortex, which normally expresses Pax6, but not in ventral telencephalic regions where Pax6 is not expressed. As in the thalamus, *Robo2* is expressed by cells in the Pax6 non-expressing cortical mantle layer rather than in the Pax6 expressing cortical ventricular zone, indicating that within both the cortical and thalamic lineages the autonomous regulation of *Robo2* by Pax6 in postmitotic neurons does not occur by direct binding of Pax6 to *Robo2* regulatory elements. Most likely is that Pax6 initiates changes in the levels of other transcription factors in the progenitors and it is the persistence of these changes in the postmitotic progeny that influence *Robo2* expression. In the thalamus, Nkx2.2 might be one such transcription factor. It will be interesting in the future to gain a more comprehensive picture of the intrinsic, cell autonomous actions of this transcription factor in this important forebrain region.

## Conclusion

Our study provides new information on the intrinsic actions of Pax6 within the thalamic lineage. Our results indicate that Pax6 is required by thalamic progenitors for the normal molecular patterning of the thalamic neurons that they generate. However, thalamic neurons can grow axons that can be guided appropriately without the need for normal Pax6-dependent patterning. In this region, normal Pax6-induced molecular patterning of neurons is not a prerequisite for their successful development of axons.

## Methods

### Mice

All animal husbandry was conducted in accordance with the UK Animals (Scientific Procedures) Act 1986. The work was approved by the University of Edinburgh’s Veterinary Ethical Review Committee leading to the award of Project Licence (60/4545) by the Home Office (UK). To create *Pax6* null embryos we used the *Pax6*^*Sey*^ allele (designated *Pax6*^*−*^) [44]. To conditionally inactivate Pax6 we used the *Pax6*^*loxP*^ allele [18]. For the staging of embryos, midday on the day of vaginal plug detection was considered as embryonic day 0.5 (E0.5). Cre expression was induced in *CAGG*^*Cre*^; *Pax6*^*loxP/loxP*^ embryos with 10 mg tamoxifen (Sigma) administered by oral gavage of pregnant females at a concentration of 50 mg/ml.

To obtain *Pax6*^*+/+*^↔*Pax6*^*−/−*^ chimeric embryos, *Pax6*^*−/−*^ embryonic stem cells which carried one copy of the TP6.3 tau-GFP transgene [45, 46] were injected into blastocysts from C57BL/6 × CBA crosses [10]. Blastocysts were then transferred to the uterus of pseudo-pregnant females and were allowed to develop. Resulting chimeric embryos express tau-GFP in all cells that originate from *Pax6*^*−/−*^ embryonic stem cells.

### Immunohistochemistry

For embryos aged E12.5 to E15.5 heads were removed and fixed in 4 % paraformaldehyde (PFA) in phosphate buffered saline (PBS) overnight at 4 °C. For embryos aged E16.5 to E18.5 the whole brain was removed and then fixed as above. Heads/ brains were either embedded in paraffin wax, or embedded in 4 % agarose, or cryoprotected by immersion in 30 % sucrose in PBS and embedded in OCT. Wax sections or cryo-sections were incubated with following primary antibodies: mouse anti-Lhx1/5, mouse anti-Nkx2.2 and mouse anti-Pax6 (all 1/200, DSHB), rabbit anti-GFP and anti-Prox1 (both 1/1000, Abcam), rat anti-L1 (1/500, Millipore) and rabbit anti-Sox2 (1/200, Ab5603 Chemicon).

### In situ hybridisation

In situ hybridizations for *Slit1*, *Slit2*, *Robo1* and *Robo2* were performed on 100 μm agarose embedded sections using digoxigenin labelled antisense riboprobes as previously described [47].

### Axon tract tracing

Whole brains were dissected between E13.5 and E18.5 and fixed for at least 48 h with 4 % PFA in PBS at 4 °C. After fixation brains were washed with PBS. For thalamic injection the brains were cut in half at the midline in the sagittal plane and a small hole was made in the medial aspect of the thalamus using a fine probe. Crystals of DiI (Invitrogen) were inserted into the prepared hole in the thalamus. For cortical or ventral telencephalic injection, holes were made in the desired region of the telencephalon without any further dissection of the brain and crystals of either DiI or DiA were inserted. Brains were incubated in PBS at 37 °C. E14.5 brains were incubated for 1 week while E16.5 and E18.5 brains were incubated for 3–4 weeks. After diffusion the brains were embedded in agarose and sectioned either coronally (telencephalic injections) or at a 45° angle (thalamic injections) at 100 μm. Sections were counterstained with TOPRO-3 diluted 1/1000 in PBS.

### Quantitative reverse transcription-PCR (qRT-PCR)

Tissue samples from the thalamus and hypothalamus of *Pax6*^*+/+*^ and *Pax6*^*−/−*^ embryos were collected and flash frozen on dry ice. Total RNA was extracted using an RNAeasy Mini kit (Qiagen), cDNA was created using Superscript reverse transcriptase (Invitrogen) and quantitative reverse transcription PCR (qRT-PCR) analysis was then carried out using a Quantitect SYBR Green PCR kit (Qiagen) with the following primer pairs: *Slit1* (5′-CCTGCCAGATGATCAAGTGC-3′ and 5′-GCTGCTTCTGGTAATAGTCC-3′), *Slit2* (5′-TCACTGACCTGCAGAACTGG-3′ and 5′-ACCATCTGGTCGAAGGTGAC-3′), *Robo1* (5′-GCCACTTCCATGCCTCTCAG-3′ and 5′-GTGCCTTGGACTGGACAGTG-3′), *Robo2* (5′-GCAGAAGTAAACCGGACGAA-3′ and 5′-CTCCAAGATTGCAGGCTCTC-3′). The abundance of each transcript (relative to *GAPDH*) was calculated.

## Abbreviations

RT-PCR: Reverse-transcription PCR
Cx: Cortex
DTB: Diencephalic-telencephalic boundary
E(n): Embryonic day n
GE: Ganglionic eminence
GFP: Green fluorescent protein
Hip: Hippocampus
Hy: Hypothalamus
PBS: Phosphate buffered saline
PFA: Paraformaldehyde
PSPB: Pallial-subpallial boundary
PTh: Prethalamus
TCA: Thalamocortical axon
Th: Thalamus
vLG: Ventral lateral geniculate nucleus
vTel: Ventral telencephalon.
WT: Wild-type
